# AI-Augmented Co-Design in Healthcare: Log-Based Markers of Teamwork Behaviors and Collective Intelligence Outcomes

**DOI:** 10.3390/bs15121704

**Published:** 2025-12-09

**Authors:** Yue Jiang, Jing Chen, Zhaoqi Li, Long Liu, P. John Clarkson

**Affiliations:** 1 College of Design and Innovation, Tongji University, Shanghai 200082, China; yue.jiang@tongji.edu.cn (Y.J.);; 2 Department of Engineering, University of Cambridge, Cambridge CB2 1PZ, UK; 3 School of Nursing and Health Management, Shanghai University of Medicine and Health Sciences, Shanghai 201318, China

**Keywords:** human-AI collaboration, medical co-design, organizational behavior, collective intelligence, teamwork behaviors

## Abstract

Co-design in healthcare settings requires teams to utilize each other’s knowledge effectively, but practical guidance and simple methods for observing collaboration are often lacking. We tested whether a lightweight AI assistant that guides the process—and automatically logs who speaks, when, and how work progresses—can make teamwork easier to manage and easier to track. Six four-person teams completed the same five-phase session. The assistant nudged timing, turn-taking, and artifact hand-offs; all interactions were recorded in a shared workspace. We assessed usability and acceptance, expert-rated product quality (technical performance), perceived team performance, and self-rated technical contribution, and we summarized basic log signals of participation and pacing (e.g., turn-taking balance, average turn duration). Analyses were descriptive. All teams finished the protocol with complete logs. Outcomes were favorable (expert ratings averaged 4.18/5; perceived performance 6.14/7; self-rated contribution 4.08/5). Teams with more balanced participation and clearer pacing tended to report better performance, whereas simply having more turns did not. A process-guiding AI assistant can quantify teamwork behaviors as markers of collective intelligence and support reflection in everyday clinical co-design; future work will examine the generalizability of these findings across different sites.

## 1. Introduction

Medical co-design, as used here, refers to collaborative design in the medical domain. In such projects, a project sponsor (organizer) brings together clinicians and designers and, when appropriate, engineers, patients, or industry partners to create human-centered responses to clinical needs (e.g., products, services, or information systems), most often through structured workshops (Bate & Robert, 2006; Greenhalgh et al., 2019; Jones, 2013; Slattery et al., 2020). We view these efforts through the lens of collective intelligence, understood as the team’s capacity to integrate the knowledge of different individuals and apply it effectively. Our focus is on small, task-focused clinical teams, which differ from public-health co-design at the community or population level, where the emphasis is on prevention and social determinants (Vargas et al., 2022). Foundational work on co-creation and design provides the conceptual backdrop for this framing (Cross, 2007; Sanders & Stappers, 2008).

Success in such settings rarely depends on a single expert. Instead, it turns on whether the group reliably displays properties typically associated with collective intelligence: timely sharing and reuse of information, complementary role taking, balanced participation, effective pacing, and progressive convergence on decisions of sufficient quality (Jiang et al., 2025a; Riedl et al., 2021/2022; Suran et al., 2021; Woolley et al., 2010). In routine practice, however, these properties are often left to the tacit skills of facilitators and the goodwill of participants. Consequently, co-design sessions may drift or stall, or become dominated by a few voices. Even when a session appears to go “well”, it is difficult to reproduce and evaluate it in a way that supports organizational learning and methodological refinement (Choi et al., 2022; Donetto et al., 2014; Lavallee et al., 2019).

This study examines whether simple, AI-supported process guidance can make it easier to measure and improve teamwork in such sessions. Rather than treating facilitation as an art form that lies outside the scope of measurement, we align session guidance with internal mechanisms known to support collective intelligence in collaborative work, including information flow, role complementarity, pacing and regrouping, and decision convergence (Jiang et al., 2025a, 2025b). The goal is not to automate creativity, but to make the conditions for collective intelligence more visible and adjustable in situ, and to make the effects of facilitation more assessable afterwards. To this end, we use a five-phase co-design prototype (v0.3) that structures multi-party sessions into a five-phase procedure, standardizes basic artifacts and prompts, and records lightweight traces of collaboration. Around this prototype, we assembled a field-ready package consisting of instruments for usability and acceptance (SUS, PU, and PEOU), team-level outcomes (TP, PP, and S-TP), and a simple logging framework that captures key steps of collaboration, such as timelines, contributions by role, and artifact evolution.

Two gaps motivate this package. First, much of the co-design literature presents successful cases or conceptual frameworks but offers little operational guidance on how to recognize, in real time, whether conditions relevant to collective intelligence are improving or deteriorating, and how to steer the process accordingly (Brandt et al., 2013; Kleinsmann & Valkenburg, 2008; Sanders & Stappers, 2008, 2014; Steen, 2013). Second, evaluation often focuses on end-of-session artifacts or satisfaction ratings, leaving the process itself under-instrumented. Without process-level signals, organizations cannot determine which facilitation moves were consequential, nor can they reliably transfer learning across settings (Choi et al., 2022; Donetto et al., 2014; Green et al., 2020; Lavallee et al., 2019; Tsianakas et al., 2012; Ward et al., 2018). Our approach addresses both gaps by combining mechanism-informed facilitation with measures that are deployable in realistic environments, thereby yielding interpretable feedback for facilitators and accumulating comparable evidence across projects.

Beyond system design, recent work calls for examining AI as a social actor and characterizing machine behavior at scale, which underscores the need for fieldable measures of human–AI teaming (Rahwan et al., 2019). In parallel, emerging agendas on hybrid human–AI collaboration argue for treating AI as a teammate and structuring interaction patterns accordingly, rather than regarding it solely as a tool (Dellermann et al., 2019; Seeber et al., 2020).

In this paper, we report a field pilot study with 24 participants organized into six four-person teams. All teams followed the same five-phase procedure, using standardized materials supported by a prototype for multi-human and multi-agent collaboration. The study was designed to assess feasibility rather than to establish causal effects: we focused on procedural adherence across teams, successful data capture, and interpretable distributions for usability/acceptance and team outcomes, complemented by process-level visualizations of participation balance, session pacing, and information reuse. Analyses are descriptive (phase-level comparisons and correlations) and are intended to inform practical iteration rather than to adjudicate specific causal models.

This paper provides three practical contributions. First, it introduces a toolkit for medical co-design in real-world settings, including a language-model assistant that guides timing and turn-taking, an automatic log of key steps, and a concise set of transparent measures. Second, it reports feasibility findings from a six-team study, covering usability and acceptance alongside team outcomes, each summarized with 95% bootstrap confidence intervals. Third, it shows that simple process signals—such as more balanced participation and fewer elongated or fragmented timelines—tend to co-occur with higher perceived performance, and it translates these signals into concrete facilitation routines (e.g., targeted prompts, time-boxing).

## 2. Related Work and Mechanism-Informed Rationale

### 2.1. Key Concepts and Definitions

Medical co-design in this paper refers to a structured, participatory design approach in healthcare settings, in which clinicians, patients (or their proxies), and designers work together to co-create concepts within clinical, ethical, and organizational constraints (Bate & Robert, 2006; Greenhalgh et al., 2019; Jones, 2013; Slattery et al., 2020). Our focus is not on any specific branded methodology, but on how concrete practices shape interaction among participants.

We define collective intelligence as a team-level capacity to integrate distributed knowledge through coordinated interaction, thereby achieving outcomes that are superior to what individuals could produce alone (Jiang et al., 2025a; Riedl et al., 2021/2022; Suran et al., 2021; Woolley et al., 2010). In this study, collective intelligence is observed through two complementary lenses: expert ratings of team products (TP) and participants’ perceptions of collaboration and contribution (PP, S-TP). We also distinguish orchestration from facilitation. Facilitation typically concerns what teams discuss or decide, whereas orchestration focuses on when and how teams interact—structuring sequences of activities, pacing work through time-boxes, guiding turn-taking via role rotation, and prompting artifact hand-offs—while remaining agnostic about content. The present study uses orchestration only, as it produces observable process traces that are well-suited for analysis.

The AI support in this work is explicitly scoped to process assistance. A language-model-based Design Service Agent (DSA) delivers short natural-language prompts related to timing, turn-taking, role rotation, and hand-offs, and records timestamped actions in a shared workspace (DIMS). Within DIMS, participants may, if they wish, invoke creative assistants for ideation. Such usage is logged, and these assistants are not used to provide medical advice or definitive design recommendations.

Throughout the paper, we use the term “markers” to denote observable process features derived from interaction logs—such as the number of dialogue turns, total and average turn duration, per-role turn counts, and the balance of turn-taking. In Section 5, we relate these markers descriptively to team outcomes.

### 2.2. Collective Intelligence in Co-Design: Internal Mechanisms

Prior research suggests that group performance depends not only on the abilities of individual members, but also on the quality of their interaction—how information flows, how roles complement one another, how pacing is managed, and how groups converge on shared decisions (Boimabeau, 2009; Jiang et al., 2025b; Malone et al., 2010; Radcliffe et al., 2019; Reia et al., 2019; Suran et al., 2021; Woolley et al., 2010). Early experimental work identified a latent collective intelligence factor that predicts team performance across multiple tasks, shifting the focus from individual traits to emergent, interaction-level properties (Woolley et al., 2010). Subsequent studies formalized multi-level quantification strategies and argued for pairing behavioral outputs with multimodal process signals to characterize collective intelligence during collaboration (Almaatouq et al., 2021; Malone & Woolley, 2020; Mao et al., 2016; Riedl et al., 2021/2022). Reviews have further synthesized mechanisms that shape collective intelligence—such as information-exchange structures, feedback loops, and cognitive diversity—emphasizing that higher collective intelligence reflects the organization of interactions rather than a simple aggregation of abilities (Jiang et al., 2025a; Salminen, 2012; Suran et al., 2021; Zhang & Mei, 2020).

Within co-design, these mechanisms align naturally with health-system design practice. Information sharing is expressed through artifact reuse and cross-referencing; role complementarity appears in rotating facilitation, synthesis, and evaluation functions; pacing is influenced by time-boxing and regrouping; and convergence is achieved through iterative framing and resolution (Chmait et al., 2016; Jiang et al., 2025a, 2025b; Kittur et al., 2009). In clinical and organizational contexts, considering these mechanisms through a human-centered and inclusive lens also foregrounds the importance of equitable participation and adherence to safety constraints.

**Figure 1:**
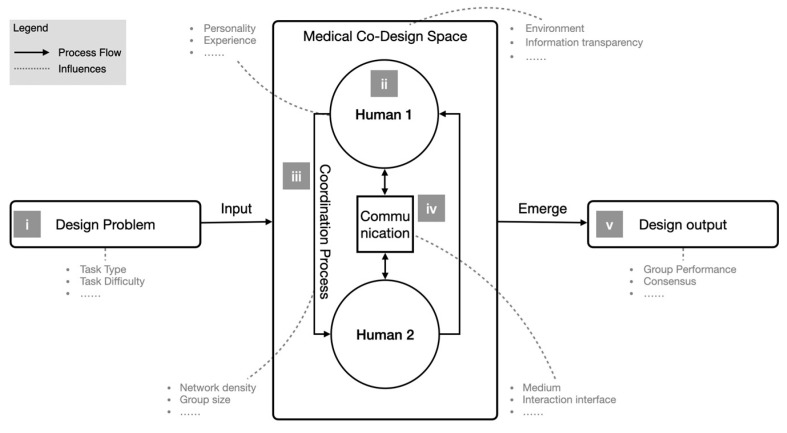
Interaction flow and factors shaping collective intelligence in medical co-design (Jiang et al., 2025a, 2025b).

Figure 1 consolidates our earlier schematics (Jiang et al., 2025a, 2025b) and clarifies the interaction flow and factors that shape collective intelligence in medical co-design (Amelkin et al., 2018; Malone & Woolley, 2020; Riedl et al., 2021/2022; Woolley et al., 2010). For clarity, the figure depicts two actors (the primitive unit in our context) and is organized into five elements: (i) the design problem entering the medical co-design space; (ii) individual (human 1/2) and team factors within that space; (iii) coordination processes between partners inside the co-design space; (iv) communication patterns that shape interactions; and (v) emergent phenomena and outputs that reflect collective intelligence at the team level. Solid arrows indicate the main process flow (inputs → co-design space → emergence → outputs), whereas dashed arrows indicate sub-influences. We use this descriptive schematic both to align orchestration with internal mechanisms and to delimit the scope of instrumentation (SUS/PU/PEOU; TP/PP/S-TP; lightweight logs of phase timing, role participation, and artifact linking).

### 2.3. Facilitation in User-Centered Collaborative Design

User-centered design provides a substantial tradition of facilitation practices and co-design frameworks; however, many accounts place greater emphasis on outcomes or case narratives than on in situ guidance—especially in health system contexts—regarding how to shape the interaction process as it unfolds (Cardoso & Clarkson, 2012; Dong et al., 2004; Goodman-Deane et al., 2010; Moss et al., 2023). Existing reviews often describe governance structures, roles, and artifacts in detail but offer less insight into how facilitators interpret process signals and adjust pacing or information surfacing in real time (Bevan Jones et al., 2020; Greenhalgh et al., 2019; Slattery et al., 2020; Sumner et al., 2021).

In this work, we distinguish facilitation from orchestration. Facilitation typically focuses on what teams discuss or decide (for example, eliciting needs or reframing problems). Orchestration, in contrast, structures when and how teams interact—sequencing activities, enforcing time-boxes and role rotation, and prompting artifact hand-offs—while remaining agnostic about content. Our study concentrates on orchestration because it yields observable process markers and can be executed by a rule-based controller that does not generate domain content. In practice, this approach translates facilitator intent into adjustable prompts, role swaps, and time structures that are aligned with mechanisms relevant to collective intelligence, such as participation balance, dependency-aware pacing, and decision convergence. This stance is consistent with behavioral evidence that trust in automation depends on perceived reliability and clarity of roles, which process-level orchestration can help make explicit (Hancock et al., 2011).

**Figure A1:**
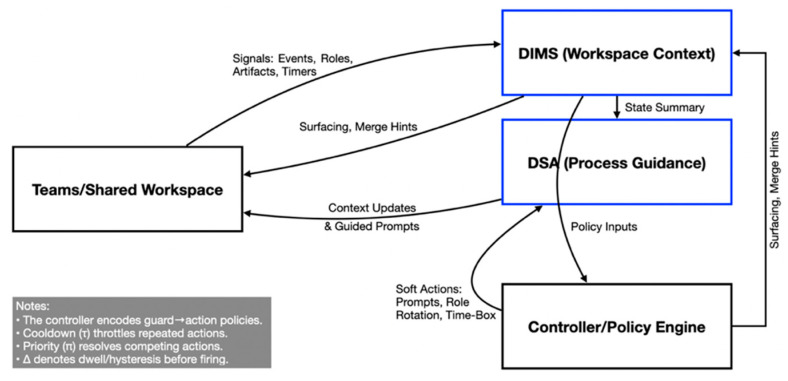
Controller flow and its placement within DSA/DIMS (Δ: dwell; τ: cooldown; π: priority).

Operationally, we employ a small set of reusable rules. For example, if participation remains skewed over a short dwell period, the system issues a prompt directed at a quieter role; if a dependency has been cleared but no new action begins within that dwell period, the system nudges the next step. The controller evaluates such guards against the live session state and produces short natural-language prompts or phase transitions, while logging all events for post hoc analysis. It operates solely on workspace state—event metadata such as timestamps, roles, phase IDs, action types, counters, and status flags—and emits only brief process prompts; it does not access artifact text or images and does not generate clinical or design content. An architectural summary is provided in Appendix C (Figure A1), where formal symbols (Δ dwell time, τ cooldown, π rule priority) are defined on first use. These rules encode facilitation expertise in an operational form while remaining compatible with routine governance and data-protection constraints (Kephart & Chess, 2003; Widom & Ceri, 1995).

### 2.4. Practical UX and Team-Level Measures in Co-Design

This section describes the practical toolkit used in the study, which combines SUS, PU, and PEOU for assessing usability and acceptance with team-level outcomes TP, PP, and S-TP. In parallel, an automatic event log records timestamps, roles, phases, action types, and artifact references (Clarkson, 2022; Clarkson et al., 2013, 2017). The package runs on standard laptops with a shared workspace and does not require any additional system integration.

First, usability and acceptance are measured using the System Usability Scale (SUS) and Technology Acceptance constructs—Perceived Usefulness (PU) and Perceived Ease of Use (PEOU)—all of which are widely used in HCI. SUS is scored by re-centering odd and even items and multiplying the resulting sum by 2.5 to yield a 0–100 score (Equation (1)) (Bangor et al., 2008; Brooke, 1996).

(1) $$S\;=\;2.5\sum_{i=1}^{10}\left[\frac{1-{\left(-1\right)}^{i}}{2}\;\left(s_{i}-1\right)+\frac{1+{\left(-1\right)}^{i}}{2}\;\left(5-s_{i}\right)\right],$$

where $s_{i}$ is the raw item score and $S\in \left[0,\;100\right]$. We adopt standard Technology Acceptance Model (TAM) definitions for PU and PEOU (Davis, 1989). In our prior deployment, different Likert anchors (0–5 and 0–7) were linearly rescaled to canonical ranges prior to aggregation; the same standardization scripts and data schema are reused here to support reproducibility.

Second, team-level outcomes comprise Technical Performance (TP), Perceived Performance (PP), and Self-rated Technical Performance (S-TP). TP reflects expert ratings of output quality and technical attainment; PP captures participants’ subjective assessment of team collaboration and outcomes; S-TP records each member’s self-rated technical contribution. A unified scale and item structure ensure cross-source comparability, and the same standardization and ID-cleaning pipeline is used to enable replication.

Third, process logging provides observables that are aligned with mechanisms relevant to collective intelligence—participation balance, coordination latency, information reuse, and convergence cues—without interfering with ongoing work. The logs record timestamped actions, actor roles, phases, artifact references, and dependency events.

Table 1 summarizes the instruments and their operationalization, including items and scales, scoring, and standardization procedures. And in the two right-most columns, this table shows (i) analytical use and (ii) conceptual role in the study (item texts are provided in Appendix A and Appendix B).

Table 2 lists the event-log fields—timestamp, actor_role, phase_id, action_type, artifact_id, and dependency—and shows how each field reads out team behaviors linked to collaboration quality (e.g., pacing, participation balance, convergence trajectory, information sharing/reuse, coordination latency) used in the visual diagnostics in Section 5. The table defines an analysis-ready export schema; platforms may log these fields directly or map them from native traces. The mapping is implementation-specific and does not constrain backend design.

The log fields allow us to observe simple teamwork behaviors: pacing (how quickly a session progresses through steps), participation balance (whether speaking and turns are shared across roles), convergence trajectory (whether work narrows toward a decision over time), information sharing/reuse (how often earlier notes or artifacts are brought back into play), and coordination latency (how long hand-offs between dependent steps take).

These design choices are conservative. We rely on widely used instruments, reuse a previously tested scoring workflow and scripts, and capture only those process-log fields needed to derive the observables reported here—SUS/PU/PEOU distributions, TP/PP/S-TP aggregates, RTC stacks, and associated correlations. This keeps instrumentation low-burden and reusable while maintaining compatibility with routine governance and data-protection practices. The measurement emphasis also aligns with organizational findings that intention and acceptance shape transformation outcomes in AI-enabled workplaces (T.-J. Wu et al., 2025).

### 2.5. Organizational Behavior Lens for Human-AI Collaboration

Building on our prior CoX framework (Jiang et al., 2025b), which separates external conditions from the interaction mechanisms that shape teamwork, we view small co-design teams as groups whose collective performance improves when interactions are structured. Here, “structured” means that timing and progression follow a few simple rules that keep work moving toward a decision (e.g., clear phases start and end, and prompts that help resume stalled discussions).

Based on this framework and our v0.3 DSA/DIMS prototype, the AI acts as a process guide. It opens and closes phases, presents the next step only after the current one is acknowledged as complete, and records each transition and prompt. When a discussion idles for a while (no input detected), it posts a short nudge to summarize or continue. These content-agnostic controls make sessions easier to run consistently, and the resulting event traces (phase changes, prompts, timestamps) enable us to quantify behavior in Section 5.

From an organizational-behavioral angle, such processes align with evidence that employees’ intentions are pivotal in shaping the outcomes of digital-intelligence transformation, reinforcing our choice to pair usability/acceptance (SUS, PU, PEOU) with mechanism-readouts (T.-J. Wu et al., 2025). Clear role boundaries and lightweight nudges are also relevant controls, as recent work shows that human–AI collaboration can influence employees’ cyberloafing via AI-identity processes (Xu et al., 2025).

## 3. Materials and Methods

### 3.1. Research Co-Design Prototype (v0.3): Multi-Agent Collaboration Support

The platform couples a rule-based Design Service Agent (DSA) with a shared workspace (DIMS). The DSA issues short natural-language prompts for timing, turn-taking, role rotation, and artifact hand-offs and records timestamped actions, while DIMS hosts shared artifacts and the interaction log. The system orchestrates process-level structure while remaining content-agnostic. Within DIMS, participants can optionally invoke creative assistants that generate non-clinical artifacts for ideation (e.g., brief needs summaries, concept images, short videos). All invocations are logged (including tool, time, and requesting role), and outputs are stored alongside team artifacts. The AI is strictly limited to process guidance and procedural Q&A and does not provide clinical advice or design proposals.

We used a research co-design prototype (v0.3) to support multi-party collaboration in medical design tasks. This prototype is used to support and complement the five-phase procedure for conducting workshops by the teams. Section 4.2 provide detailed information on the phase-by-phase orchestration, team agreements, instruments, and log markers. The prototype delivers role-aware prompts and templates and logs process traces without interrupting work.

The controller schedules prompts and transitions using rules aligned with CI-supporting mechanisms, encouraging balanced participation, timely regrouping, and progressive convergence, while the workspace consolidates artifacts and references. A log collector records timestamps, actor roles, phase IDs, action types, and artifact references, with privacy filters and de-identification applied at export to enable post hoc visualization of pacing and role contributions (Section 5). Privacy filters redact sensitive free-text (e.g., notes, files, screenshots) before logging or export, and de-identification replaces direct identifiers with pseudonymous tokens or a null value in the exported file; both steps are applied before analysis.

**Figure 2:**
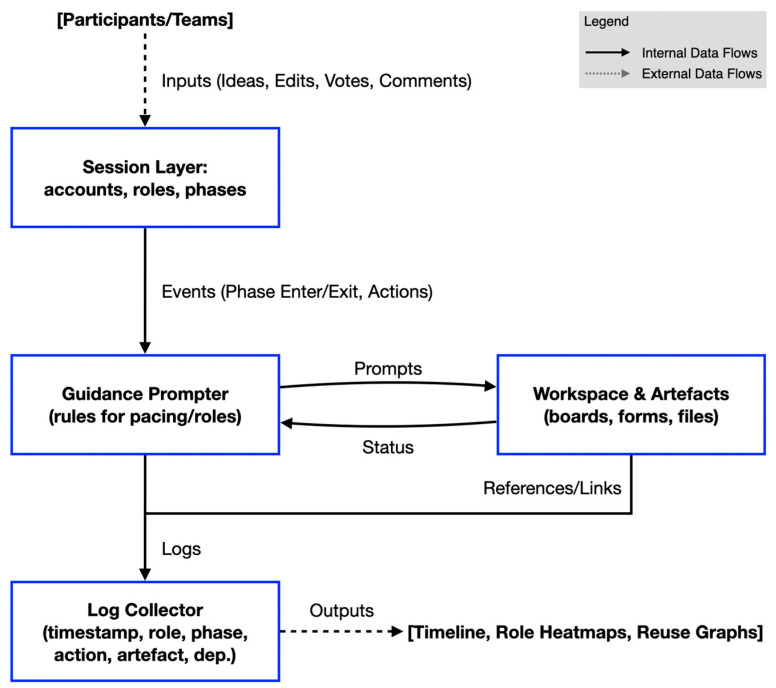
Research co-design prototype (v0.3): components and data flow. The Session layer emits events (phase enter/exit, actions). The process-guidance controller (DSA) sends prompts to the workspace and receives status updates; the workspace hosts shared artifacts. A log collector records logs (timestamp, role, phase, action, artifact reference, dependency) and references/links for later visualization. This figure illustrates the data/interaction flows; the five phases of the procedure are described in Section 4.2.

Figure 2 shows the components and data flow of the prototype. An architecture summary (Appendix C) explains how the controller (DSA) interfaces with the workspace (DIMS). Here, we focus on field-ready measures and mechanism-informed facilitation relevant to health-system HCI practice.

Table 3 lists the four modules—Session Layer, Process-Guidance Controller (DSA), Workspace and Artifacts (DIMS), and Log Collector—together with their responsibilities and the core input/output fields, explicitly mapping outputs to the process-log schema in Table 2 (e.g., controller → prompt_type/target_role/content; log collector → timestamp/actor_role/phase_id/action_type/artifact_id/dependency). Table 3 also specifies only the analysis-facing exports, as Table 2 does.

The design goal is practitioner neutrality: facilitators can conduct routine sessions as usual, while the system provides just enough structure to make conditions relevant to collective intelligence observable and to support iteration toward improved pacing and participation balance.

### 3.2. Measures and Operational Definitions

We combine widely used usability and acceptance instruments with program-specific team-outcome measures and a simple process log (Appendix A and Appendix B; Table 2). All scoring and rescaling procedures follow conventions used in prior HCI/healthcare research and our earlier deployments. Conceptual definitions are summarized in Section 2.1; here, we provide detailed measurement procedures and coding rules for each marker.

#### 3.2.1. Usability and Acceptance: SUS, PU, PEOU

SUS is scored per Brooke’s convention, yielding $S\in \left[0,\;100\right]$ (Equation (1)) (Bangor et al., 2008; Brooke, 1996). Perceived Usefulness (PU) and Perceived Ease of Use (PEOU) follow TAM item sets and are aggregated as item means after linear rescaling to a canonical 1–7 range (Equation (2)) (Davis, 1989). If a questionnaire used L−U anchors other than 1–7 (e.g., 0–5), each response x is mapped to $\tilde{x}$ as:

(2) $$\tilde{x}\;=\;1+6\cdot \frac{x-L}{U-L}\;,\;\tilde{x}\in \left[1,7\right],$$

where L and U are the lower and upper anchors of the original scale.

#### 3.2.2. Team-Level Outcomes: TP, PP, S-TP

Technical Performance (TP) is expert-rated on a unified rubric (problem framing, clinical appropriateness, feasibility, coherence), averaged across raters. Perceived Performance (PP) captures participants’ appraisal of team output and collaboration quality; Self-rated Technical Performance (S-TP) aggregates each member’s self-reported contribution. Scores that use different anchors are first converted to a common range via Equation (2) so that ratings are comparable across raters and tasks.

For measurement provenance and reliability, the TP, PP, and S-TP instruments were developed in our prior work for this program; item pools and scoring rules are unchanged here. In earlier deployments, internal consistency was high (TP α = 0.953; PP α = 0.936), and S-TP shares TP items and anchors. In this article, we use them as team-outcome summaries; full construction details and additional validation analyses are reported elsewhere, with item texts provided in Appendix B.

#### 3.2.3. Process Logs: Collaboration Timelines, Role Contributions, Artifact Evolution

The log schema is designed to be lightweight, preserving privacy and session flow while enabling mechanism-aligned observables. We track pacing/latency (phase enter/exit, time between dependency satisfaction and next action), participation balance (events per role and their dispersion), and information reuse (links to prior artifacts across teams/roles/phases). Dialogue-turn metrics (NDT/TDD/ATD) were derived from a combination of exported events and light manual annotation, where turn boundaries were not explicitly defined in v0.3 logs. These exports underpin the RTC stacks and correlation views reported in Section 5; an example export is available on request (Data Availability Statement).

Table 4 summarizes the operationalization across instruments and logs—listing raw scales, scoring/aggregation (SUS via Equation (1); all other Likert anchors rescaled via Equation (2)), and the analysis outputs used in Section 5.

#### 3.2.4. Uncertainty and Reliability

Analyses are reported at the level of data collection. For bounded perception scales collected at the individual level (SUS, PU, PEOU; *n* = 5 after cleaning), we report means with 95% confidence intervals estimated using bias-corrected and accelerated bootstrap (BCa; B = 10,000 resamples) (Efron, 1987). Bias-correction and acceleration are obtained by jackknife over individuals, and intervals are truncated to legal scale ranges; medians and IQRs are shown alongside means because distributions may be non-normal in small samples. 

For team-level outcomes (PP, S-TP, and TP; *n* = 6 teams), we analogously report team means with BCa 95% CIs based on team-level resampling, with ranges truncated to the scale limits.

Expert ratings of Technical Performance (TP) also have an inter-rater component. We quantify inter-rater reliability using the two-way random-effects, absolute-agreement ICC(2,k) based on the standard ANOVA mean squares. Because the design features few teams and heterogeneous raters, ICC is treated as a diagnostic; the estimate and BCa 95% CIs are reported in the Supplement Materials (Tables S1 and S2). In the main text, we emphasize that team-level TP means with BCa CIs are used.

For associations between log-derived conversational markers—NDT, TDD, ATD, RTC-per-role, and TTB—and team outcomes (TP, PP, S-TP), we summarize Pearson’s *r* and provide 95% CIs from percentile bootstrap on Fisher’s z with back-transformation. Given the small-N and potential non-normality/ties, Spearman’s ρ with percentile-bootstrap CIs is computed as a robustness check and reported in the Supplement Materials (Tables S3 and S4) (de Winter et al., 2016). All correlations are interpreted descriptively; no null-hypothesis tests are performed. 

No imputation was used. Each endpoint uses the available teams or individuals; any missingness and protocol deviations are logged and summarized in the Supplementary Materials.

### 3.3. Lightweight Reproducibility Package and AI Use Disclosure

Appendix A, Appendix B, Appendix C and Appendix D provide the instrument items, rubric summaries, and the export schema. To support transparency and reproduction of perception and log-derived measures, we also provide a lightweight reproducibility package: (i) full item wordings and Likert anchors for SUS, PU, PEOU, PP, and S-TP, with scoring/aggregation rules; (ii) the rating rubric for team product (TP); (iii) a variable dictionary mapping raw fields to derived markers; (iv) de-identified example interaction logs (hashed IDs, jittered timestamps, and redactions); and (v) analysis scripts that compute descriptive statistics and confidence intervals (BCa for means; percentile/Fisher-z for correlations), ICC(2,k) for TP with CIs (reported in the Supplementary Materials). De-identified scripts and an example log export are available on reasonable request (see Data Availability Statement).

During manuscript preparation, the authors used ChatGPT-5 for limited language support only (translation of some parts of author-written Chinese to English, light copy-editing, and minor style alignment to journal format). The authors reviewed and edited the outputs and take full responsibility for the content.

## 4. Study Design and Procedure

### 4.1. Sites, Participants, and Roles

We conducted the study in a real-world applied setting relevant to medical innovation. To preserve anonymity while maintaining authenticity, we describe the site only in generic terms and focus on the procedure and instrumentation. Twenty-four participants were organized into six four-person teams. Teams were formed to reflect the typical multi-disciplinary composition of medical co-design—mixing domain-side stakeholders (e.g., clinical or operational) with design/engineering roles—so that information, perspectives, and artifact work could circulate in a way that resembled routine projects (Malone & Bernstein, 2015; Malone & Woolley, 2020; Riedl et al., 2021/2022; Suran et al., 2021; Woolley et al., 2010). All procedures complied with institutional guidelines. Sessions were non-clinical and of minimal risk; no patient data were involved. No personally identifiable information was collected in the logs; role identifiers were limited to coarse categories, and all exports were anonymized prior to analysis.

### 4.2. Tasks and Five-Phase Procedure Aligned with the Mechanism-Informed Rationale

All teams followed a five-phase session framework typical of co-design in health settings: (I) Orientation and Briefing, a short all-hands introduction to the study background, session goals, and core functions; (II) Access and Onboarding, where participants logged in with role-based accounts and received task materials; (III) Co-Creation, the main work stage with guided cycles of idea generation, consolidation, and evaluation; (IV) Sharing and Inter-Team Exchange, where outputs were summarized and briefly presented; and (V) Post-Session Survey, where usability/acceptance and team-outcome instruments were completed. Measurement anchors and data flows for each phase were summarized in Table 5.

This structure referred to widely used user-centered co-design practice in clinical and organizational contexts—briefing/onboarding → collaborative work → sharing → post-session survey—and was chosen to standardize pacing, artifacts, and instrument timing across teams (Cardoso & Clarkson, 2012; Dong et al., 2004; Goodman-Deane et al., 2010; Moss et al., 2023; Sanders & Stappers, 2008, 2014).

Before Phase I, all teams received a standardized physical briefing and verbal orientation that stated and confirmed the following agreements: (1) fixed time-boxes per phase with countdown timers; (2) turn-taking rules enforced by the system, including role rotation at predefined checkpoints; (3) single-threaded conversation (no side discussions); (4) artifact hand-offs via DIMS with prompts; and (5) respectful interaction and compliance with the logging protocol. Participants acknowledged these agreements before proceeding. The orchestrator then implemented the five-phase procedure; any deviations were recorded in the log and were addressed by automated prompts rather than human content facilitation.

Creative assistants within DIMS were available throughout as optional tools for ideation; their use was neither required nor restricted to specific phases of the project. Participants were reminded that these tools do not provide clinical advice and that any generated artifacts served only as stimuli for team discussion.

Prompts and templates were identical across teams, and transitions were guided by rules that supported conditions relevant to collective intelligence—balanced participation, dependency-aware pacing, and progressive convergence—while lightweight process events (phase enter/exit, action types, and artifact references) were logged without disrupting work. TP (expert) was administered after the Sharing stage; SUS/PU/PEOU and PP/S-TP were administered in the Post-Session Survey (Section 3).

### 4.3. Deployment History

**Figure 3:**
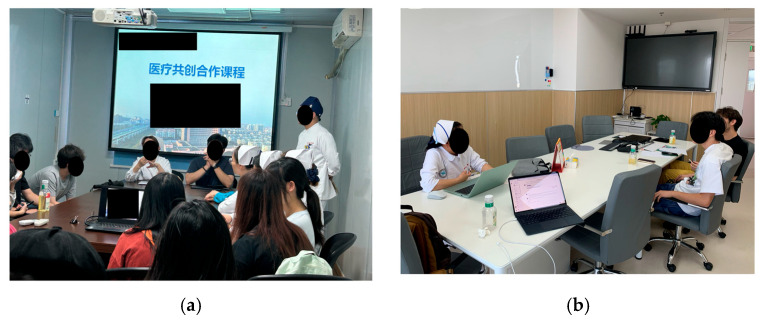
On-site photos of the medical co-design course. (**a**) Opening briefing and all-hands discussion in the seminar room; The Chinese text displayed on the screen refer to “Medical Co-Design Cooperation Course”. (**b**) Small-group work during task execution in the collaboration room. Faces and other identifying details are masked; photos were taken by the authors’ team or collaborating staff with permission.

A formative classroom walkthrough preceded the field study (shown in Figure 3). Its purpose was to validate the procedural choreography, prompt content, and artifact templates in a formative, non-evaluative setting. No systematic logs were collected; therefore, the walkthrough is excluded from analyses and retained only as design context. As a result of the walkthrough, we tightened time-boxes for Co-Creation, simplified the prompt taxonomy, and added a dependency tag to support latency diagnostics. These adjustments reduced session overhead and improved the signal quality of pacing and dependency latency metrics in the field.

**Figure 4:**
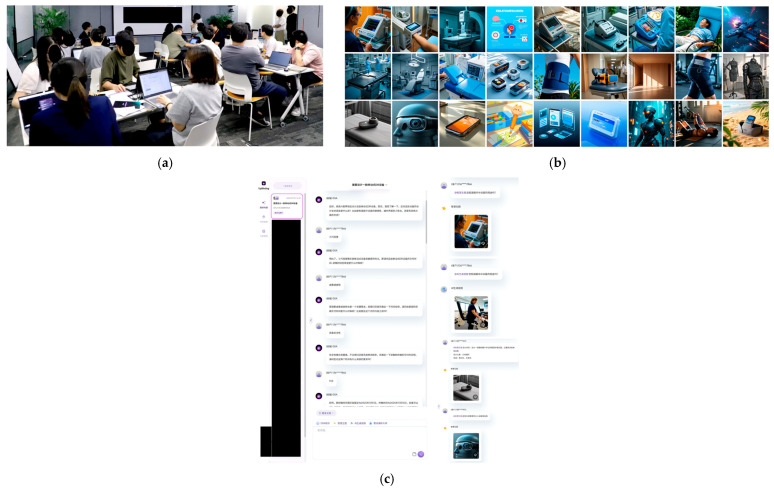
Platform context and artifacts used during the study. (**a**) Participants working in the digital workspace during co-creation. (**b**) Example concept images generated by the creative assistant and shared in the workspace for ideation. (**c**) Platform conversation/workspace screen displaying prompts, messages, and file transfers; personal information has been redacted for privacy; The Chinese text in the screenshot mainly shows the conversation process of people at the workshop using the prototype. It also includes the interface of the system itself, the names of each real person or intelligent agent character, etc.

The field study then deployed the full procedure with 24 participants in six four-person teams, each using standardized accounts, identical phases, and templates, along with on-site prompts (shown in Figure 4). This deployment targeted feasibility in realistic conditions, focusing on procedural adherence across teams, data capture as planned for SUS/PU/PEOU and TP/PP/S-TP, and maintaining clean process logs suitable for mechanism-aligned visualization. The field setting ensured that patterns we report (Section 5) reflect the interaction constraints and coordination demands of actual co-design practice rather than a laboratory abstraction.

### 4.4. Data Preparation and Analysis Plan

The main text presents only the statistics and figures necessary for the study’s objectives. Scripts and file layouts are available on reasonable request (see Data Availability Statement). Instrument responses were inspected for missingness and range errors; respondent IDs were checked against team rosters; and a single, anonymized analysis ID was assigned to each participant. SUS was scored per Brooke’s convention, yielding $S\in \left[0,\;100\right]$ using Equation (1). PU and PEOU item scores were linearly rescaled to $\left[1,\;7\right]$ using Equation (2), then averaged to obtain scale means. TP was computed from expert rubric ratings, averaged after rater-wise z-standardization; PP was rescaled to [1, 7] and aggregated as team means; S-TP retained its native [1, 5] anchors and was aggregated to a team mean (Section 3.2). Process events were exported to CSV, filtered for duplicates, and aligned to phase intervals to support the construction of timelines, role-contribution heatmaps, and information-reuse graphs.

Analyses emphasized description and correlation, consistent with our feasibility aim and the state of the literature on collective intelligence in naturalistic collaboration. We report distributions (mean, standard deviation, median, interquartile range) for SUS, PU, PEOU; summary statistics for TP, PP, and S-TP; and phase-level coverage and durations. Correlations between process observables—dialogue volume, total duration, average turn duration, participation balance, and role-specific turn count—and team-level outcomes (TP, PP, S-TP) were examined using Pearson correlations. Results are interpreted descriptively, given the small-N and non-controlled design. Planned diagnostics, such as artifact-reuse density and decision latency, require richer logs and are reserved for future iterations. Because teams shared an identical procedure but worked independently, the tests are interpreted as exploratory and non-causal; the results inform design directions for the next prototype iteration rather than adjudicating among competing causal models.

Team products were scored during the workshop’s plenary session using a common rubric; raters did not have access to PP/S-TP responses while scoring. No imputation was performed; analyses use available observations, and any missingness or protocol deviations are logged and summarized in the Supplementary Materials. For outcomes on bounded scales, we report means with bootstrap confidence intervals (BCa) at the appropriate analysis level. Inter-rater reliability for TP is quantified as ICC(2,k) with 95% CIs and reported in the Supplement (Section S1.2). Correlations between log-derived markers and outcomes are summarized with Pearson’s *r* and bootstrap CIs in the main text (Section 5.5), with Spearman’s ρ and bootstrap CIs provided as a robustness check in the Supplement (Section S1.3). All perception scales and log-derived markers are linked to item/field definitions in Section 3.2 and the Appendix A, Appendix B, Appendix C and Appendix D.

## 5. Results

### 5.1. Adherence and Completion

We first report procedural adherence, instrument completion, and deliverables before turning to distributions and correlations. All six teams completed the full five-phase procedure within an approximately 90-min session, producing the required deliverables (problem-framing notes, option sets, consolidated clusters, decision artifacts). The 90-min duration reflected site logistics and participant scheduling rather than a theoretical constraint; phase time-boxes were preset to fit this window, and analyses do not depend on the absolute session length.

Instrument completion after cleaning was: SUS/PU/PEOU, *n* = 5 valid responses (from 7 collected); TP, *n* = 12 valid expert ratings (from 16; four removed due to duplication/missingness or all-zero strings); and PP/S-TP, *n* = 12 valid responses.

Process logging covered the entire session for all teams. One team initiated two runs in the system, with the first low-quality run excluded and the second retained, resulting in six valid team logs for analysis. This met the feasibility targets and enables mechanism-aligned analyses reported below.

### 5.2. Usability and Acceptance

Table 6 summarizes System Usability (SUS, 0–100) and TAM measures (Perceived Usefulness, PU; Perceived Ease of Use, PEOU; both rescaled to 1–7 per Equation (2)). Given the small cleaned participant sample (*n* = 5), we report mean ± SD together with medians and interquartile ranges (IQR), and we add 95% confidence intervals estimated via bias-corrected and accelerated bootstrap (BCa; B = 10,000) to reflect sampling uncertainty. SUS centers around common “good” thresholds (mean = 69.20, BCa 95% CI = 60.00–80.00; median = 66.00, IQR = 16.00). PEOU is high (mean = 5.63, CI = 4.71–6.43; median = 5.57, IQR = 1.14), whereas PU is moderate-to-high with wider dispersion (mean = 4.89, CI = 3.86–6.20; median = 4.14, IQR = 2.00).

**Figure 5:**
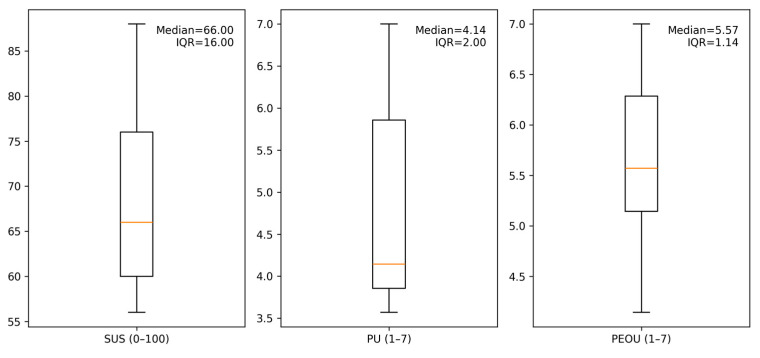
Distributions (box plots) of SUS, PU, and PEOU with medians and IQRs annotated (original scales). The horizontal orange line within each box indicates the sample median.

These patterns align with participant comments that standardized prompts reduced coordination overhead, while task-specific value could be surfaced more explicitly for certain workflows (Bangor et al., 2008; Brooke, 1996; Davis, 1989). Figure 5 shows box plots on the original scales with medians and IQRs annotated.

Reported BCa CIs indicate ranges of plausible population means under conditions of small sample size and potential skew. Given *n* = 5, intervals are interpreted as an orientation for design decisions rather than as hypothesis tests.

### 5.3. Team-Level Outcomes

Team outcomes for expert-rated Technical Performance (TP), Perceived Performance (PP), and Self-rated Technical Performance (S-TP) are summarized in Table 7 on their raw scales, with normalized means of 0–1 shown for cross-scale comparability. The ordering remains consistent—PP highest, TP next, S-TP lowest—matching the common pattern that collaborative appraisals tend to exceed individual self-appraisals, with expert judgments in between.

**Figure 6:**
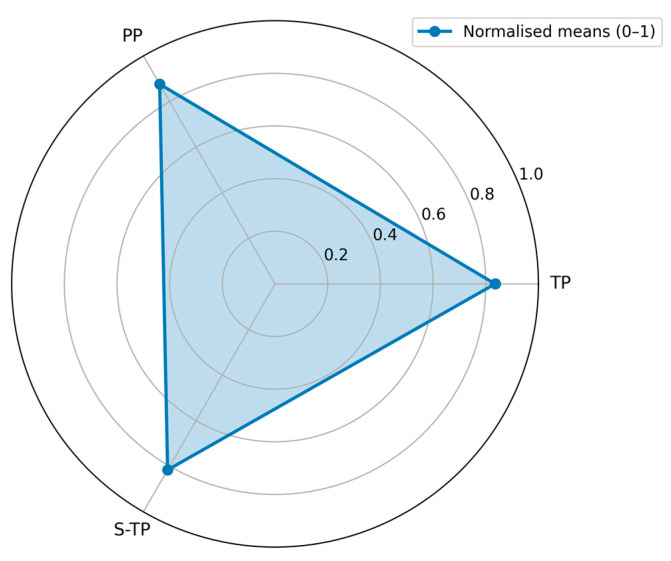
Radar plot of TP, PP, and S-TP (0–1 normalized). PP lies closest to the outer rim, followed by TP and then S-TP.

TP averaged 4.18/5 at the team level with a narrow 95% BCa CI of 4.14–4.22, indicating generally high product quality in this sample. PP averaged 6.14/7 (95% BCa CI 5.60–6.67), while S-TP averaged 4.08/5 (95% BCa CI 3.57–4.57). Intervals were estimated via bias-corrected and accelerated bootstrap (B = 10,000) at the team level and interpreted descriptively given the small number of teams. Figure 6 visualizes the normalized means; PP extends furthest toward the outer rim, followed by TP and then S-TP, mirroring the tabulated ordering.

For completeness, inter-rater reliability for TP is reported in the Supplementary Materials as a two-way random-effects, absolute-agreement ICC(2,k) with BCa 95% confidence intervals; in the main text, we summarize team-level means with BCa CIs due to the small number of teams.

### 5.4. Process-Level Diagnostics (Visualizations)

**Figure 7:**
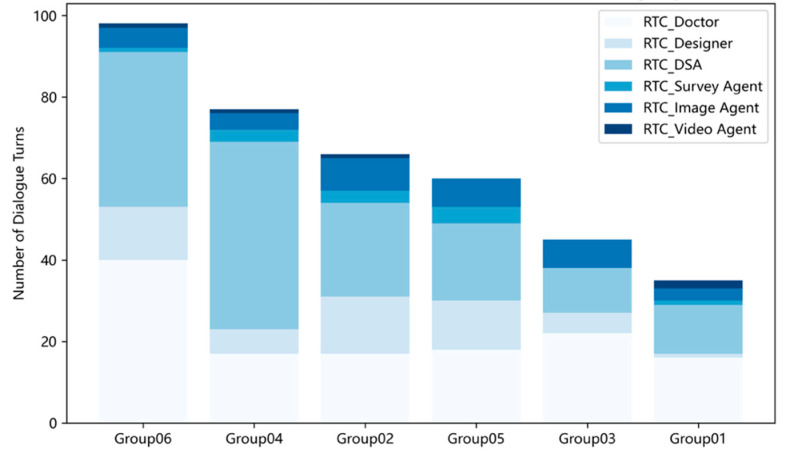
Stacked distribution of role-specific turn counts (RTC) by team. Each bar shows the composition of dialogue turns by role across the session; even mixes indicate balanced participation, whereas long single-role segments indicate dominance.

We summarize participation balance using role-specific turn counts (RTC) aggregated over the session. Figure 7 shows stacked distributions of RTC by team. Two patterns are salient. First, clinician and designer turns are relatively stable across teams, while several teams exhibit strong dominance by the process-guidance agent (DSA). For example, in Team 4, the guidance agent contributes a disproportionately large share of turns, whereas teams with higher PP/TP scores display more even role distributions. Second, auxiliary agents (e.g., research helper, media generator) intervene only sporadically, suggesting that their current triggers are conservative. These observations align with the correlation analyses (Section 5.5): more balanced participation is associated with higher perceived performance, whereas fragmented or agent-dominated exchanges are associated with lower perceived performance. We do not claim causality; the RTC view is a descriptive diagnostic to help facilitators notice when human roles are being overshadowed and when rebalancing prompts may be useful.

### 5.5. Synthesis: What Supported or Hindered Collective Intelligence Emergence in Practice

**Figure 8:**
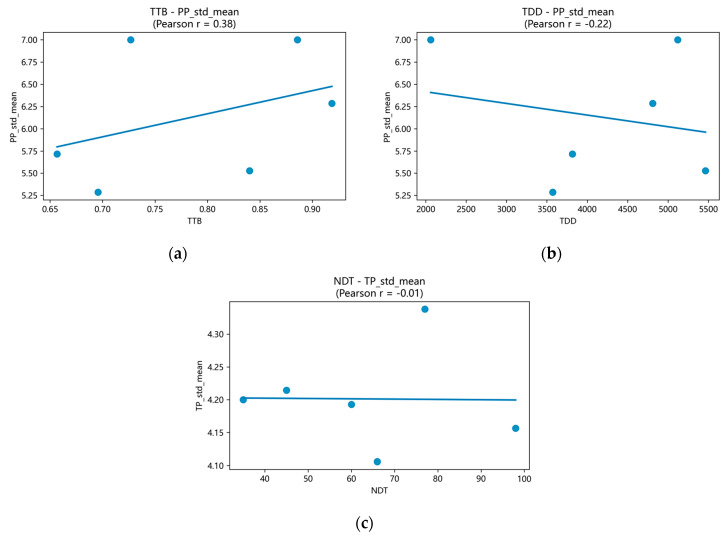
Correlation scatter examples with fitted least-squares lines: (**a**) TTB vs. PP (positive trend); (**b**) TDD vs. PP (negative trend); (**c**) NDT vs. TP (near-zero).

Correlation analyses (n=6 teams^1^) relate basic conversational features to team outcomes (Table 8; Figure 8 provides exemplars). We examined NDT (number of dialogue turns), TDD (total dialogue duration), ATD (average turn duration), RTC per role (not tabulated), and TTB (turn-taking balance), against TP, PP, and S-TP using Pearson correlations (r); given the small-N, non-controlled design, results are interpreted descriptively. Patterns are co-occurrences consistent with collective intelligence theory—organized interaction, not volume alone, is accompanied by better perceived outcomes.

- Volume vs. quality: NDT showed essentially no relation to TP (r≈−0.014); simply “talking more” did not predict expert-judged output quality.
- Pacing burden: TDD correlated negatively with PP (r≈−0.221) and more strongly with S-TP (r≈−0.750), suggesting that overlong sessions undermine perceived effectiveness and individual contribution.
- Turn structure: ATD was positively related to PP (r≈0.570), indicating that longer, more substantive turns were associated with better perceived collaboration. TTB also showed a positive relationship with PP (r≈0.377), highlighting the value of balanced participation.
- Fragmentation: A strong negative NDT–PP correlation (r≈−0.922) indicates that more fragmented turn-taking co-occurred with lower perceived performance.

Together with the visual diagnostics (Figure 5, Figure 6, Figure 7 and Figure 8), these correlations reinforce three practice-level levers: pursue balanced participation, guard against overlong timelines and fragmented micro-turns, and scaffold more substantive turns. These levers are operational within the instrumented package and map directly to facilitation moves (e.g., targeted prompts, time-boxing), offering a practical pathway to understand and steer collective intelligence in medical co-design.

To make the uncertainty of the exploratory correlations explicit, we computed 95% confidence intervals for each Pearson coefficient using a percentile bootstrap with Fisher-z transformation (B = 10,000; Table 9). With only six teams, intervals are necessarily wide and should be interpreted descriptively. Intervals spanning zero indicate that the data are compatible with both positive and negative relations in this sample, whereas intervals that remain on one side of zero suggest a directionally stable association under resampling.

Two patterns stand out. First, the fragmentation signal persisted: the relationship between the number of dialogue turns (NDT) and perceived performance (PP) was strongly negative (*r* = −0.922; 95% CI, −1.000 to −0.729). Second, longer total durations were associated with lower self-rated technical performance (S-TP) (TDD-S-TP: *r* = −0.750; 95% CI, −0.998 to −0.168). Other estimates—e.g., ATD-PP (*r* = 0.570; 95% CI −0.438 to 1.000), TTB-PP (*r* = 0.377; 95% CI −0.526 to 0.907), and NDT-TP (*r* = −0.014; 95% CI −0.984 to 0.875)—had intervals that cross zero, so we treat them as suggestive tendencies aligned with the practice levers above rather than firm effects.

A rank-based robustness check yielded the same qualitative picture (e.g., ρ = −0.928 for NDT-PP and ρ = −0.771 for TDD-S-TP; see Tables S3 and S4). Taken together, the CI analysis reinforces our practical guidance: avoid fragmented, overlong exchanges while promoting balanced, more substantive turns.

## 6. Discussion and Conclusions

### 6.1. Implications for Organizational Behavior and UCD/UX Practice

From an organizational-behavior perspective on employee-AI collaboration, our findings frame participation balance and pacing/latency as observable work behaviors that facilitation can monitor and nudge at low burden (Malone & Bernstein, 2015; Malone & Woolley, 2020). This study further shows that mechanism-informed facilitation can be implemented as concrete, observable behaviors in employee–AI collaboration, rather than remaining at the level of abstract principles.

By pairing widely used usability/acceptance instruments (SUS, PU, PEOU) with concise, openly specified team-outcome measures (TP, PP, S-TP) and low-overhead logs, facilitators obtain actionable readings of conditions relevant to collective intelligence—participation balance and pacing/latency (Almaatouq et al., 2021; Jiang et al., 2025a; Woolley et al., 2010). Interpreted descriptively and without causal claims, the patterns suggest that balanced participation and avoiding overlong or fragmented timelines co-occur with higher perceived team performance. In practice, three routines follow: (i) tie targeted prompts to participation dispersion (when dispersion worsens over a fixed window, nudge quieter roles); (ii) use time-boxing with visible latency so teams see when dependencies clear but actions stall; and (iii) scaffold turn quality with light templates (problem–evidence–proposal) to reduce micro-turn fragmentation. Because the measures and logging are fully specified and low-overhead, teams can adopt the package without workflow changes, and organizations can accumulate comparable evidence across projects in a way that aligns with established user-centered design practice (Cardoso & Clarkson, 2012; Sanders & Stappers, 2008).

Beyond feasibility, AI-mediated exchanges may also scaffold reflective reasoning: exploratory evidence suggests that chatbot interactions can boost critical-thinking skills, which motivates our use of concise templates and prompts for turn quality (Fabio et al., 2025). In parallel, AI-assistant effects can be channeled through self-efficacy mechanisms, indicating that perceived capability may modulate performance benefits from guidance (Hafeez et al., 2025). These field-deployable traces complement calls to study human–AI teams as socio-technical systems with measurable machine behavior and teamwork dynamics (Dellermann et al., 2019; Kim et al., 2024; Rahwan et al., 2019).

### 6.2. Design Directions Toward v0.4

Findings indicate several bottlenecks and suggest concrete design directions. The largest negative correlations observed in this sample involved total dialogue duration with PP and S-TP, suggesting that v0.4 should introduce adaptive pacing: when cumulative duration exceeds a phase-specific threshold without progress markers (e.g., new links, cluster merges), the system can recommend regrouping. A marked negative relation between fragmented turn volume and PP points to a need for turn-quality scaffolds—slightly longer, more substantive turns prompted by micro-templates rather than free-form chatter.

Heatmaps revealed episodes of role dominance, often by the guidance agent; v0.4 therefore can cap system-prompt frequency per window and explicitly restore attention to human roles when dominance persists to maintain role clarity and support human agency in hybrid teams (Dellermann et al., 2019; Seeber et al., 2020). Finally, to reduce manual coding overhead, v0.4 can include automatic path tracking (phase transitions, dependency clearances), richer artifact metadata (link type and intent), and a live facilitator dashboard exposing three indicators: participation dispersion, decision latency, and phase coverage/pacing.

In the longer term, affective markers could complement our low-burden logs. Deep-learning approaches to inferring emotions from facial cues are now widely applied in behavioral studies, although their use would require additional governance and consent (Telceken et al., 2025). These design moves align with routine UCD practice and are motivated by observed co-occurrences, rather than post hoc theorizing. These directions preserve human agency and role clarity in employee-AI collaboration and remain compatible with the broader literature on collective intelligence in complex teams (Elia & Margherita, 2018; Jiang et al., 2025a; Malone & Woolley, 2020).

### 6.3. Applicability and Transfer

Although our field site was an applied, non-clinical setting, the task forms (Orientation and Briefing, Access and Onboarding, Co-Creation, Sharing and Inter-Team Exchange, Post-Session Survey) are common to both clinical and non-clinical co-design (Clarkson, 2022; Greenhalgh et al., 2019; Moss et al., 2023). The package, therefore, transfers with minor adjustments. In clinical environments, governance and privacy may require stricter logging granularity and consent flows. The same low-burden schema can be further scoped (e.g., artifact type without content) while retaining the indicators above.

Team composition matters: where patient or caregiver roles are present, prompts can be tuned to protect voice (e.g., participation nudges keyed to vulnerable roles). Task types that require heavy external validation (e.g., device pathways) benefit from a stronger linkage between artifacts and evidence sources to maintain interpretable decision trails while minimizing the exposure of sensitive content. Across settings, the value proposition is stable: observable process signals allow facilitators to steer collective intelligence in situ and organizations to learn cumulatively across projects. These adjustments preserve a human-centered and inclusive stance while keeping the burden low for healthcare teams in line with established co-design traditions (Bate & Robert, 2006; Sanders & Stappers, 2014).

### 6.4. Limitations and Threats to Validity

The study is a single-site, small-N design, intended for feasibility rather than hypothesis testing. The instruments achieved full post-cleaning coverage; however, the sample does not support fine-grained modeling. The design is non-experimental; teams shared the same procedure but worked independently, so correlations are associational and may be influenced by unobserved confounding factors. Logging in v0.3 captured essential fields but remained coarse; some conversational features required manual annotation, which introduces coder judgement. Pre-specified cleaning rules excluded one low-quality system run; such decisions can bias distributions if not documented. Finally, while SUS/PU/PEOU and TP/PP/S-TP are established in HCI, anchor remapping (Equation (2)) adds a transformation layer; we mitigated this by using standard linear rescaling and reporting descriptive statistics with clear ranges (Bangor et al., 2008; Davis, 1989). These constraints are routine for naturalistic UCD studies and are addressed in the v0.4 roadmap.

While confidence intervals and ICCs improve transparency, our small sample size yields imprecise estimates and limits the generality of our findings. Self-report scales remain vulnerable to response biases; we mitigate this by triangulating with objective log-derived markers and by reporting cluster-bootstrap CIs for both estimates and correlations using established resampling procedures (de Winter et al., 2016; Efron, 1987).

### 6.5. Conclusions and Next Steps

Six four-person teams completed the protocol with full data capture. Team outcomes were favorable: Technical Performance = 4.18/5, Perceived Performance = 6.14/7, and Self-rated Technical Performance = 4.08/5. Teams with more balanced participation tended to report higher perceived performance, whereas simply having more turns did not (NDT–TP *r* ≈ −0.01; TDD–S-TP *r* ≈ −0.75; ATD–PP *r* ≈ 0.57). These observations are descriptive and provide practice-level levers for facilitation that are consistent with prior work on interaction quality and team effectiveness in collective intelligence research (Jiang et al., 2025a, 2025b; Riedl et al., 2021/2022; Woolley et al., 2010).

Next steps include implementing v0.4 with adaptive pacing, turn-quality scaffolds, automatic path tracking, and a facilitator dashboard; pre-registering evaluation plans with descriptive and robustness analyses; and conducting cross-setting replications spanning clinical wards and applied innovation teams. The broader contribution is a path for UCD/UX teams to treat understanding and steering collective intelligence as built-in capabilities, grounded in measures they already trust and signals they can observe (Almaatouq et al., 2021; Dellermann et al., 2019; Malone & Woolley, 2020).

## Figures and Tables

**Table 1:** Instruments and operationalization.

Instrument	Construct	Items and Scale	Scoring/Standardization	Analytical Use	Conceptual Role
SUS	Perceived usability	10 items; 5-point Likert	Equation (1); aggregate to 0–100	Descriptives, BCa 95% CIs	System usability of the prototype
PU	Perceived usefulness	7-point Likert	Linear rescale to 1–7; mean of items	Descriptives; BCa 95% CIs	User-perceived usefulness
PEOU	Perceived ease of use	7-point Likert	Linear rescale to 1–7; mean of items	Descriptives; BCa 95% CIs	User-perceived ease of use
TP	Technical performance (expert)	5 rubric items; 1–5 Likert; expert rating	Rater-wise z-standardization → mean across raters → back-scale to 1–5	Descriptives; BCa 95% CIs; outcome in correlations with log markers	Team-level outcome (expert-rated product quality)
PP	Perceived team performance	9 items; 1–7 Likert	Linear rescale to 1–7 if anchors differ; item mean	Descriptives; BCa 95% CIs; outcome in correlations with log markers	Team-level outcome (participant-rated performance)
S-TP	Self-rated technical contribution (per member)	5 items (parallel to TP); 1–5 Likert	Item mean per member; team aggregation by averaging members (anchored via Equation (2) if needed)	Descriptives; BCa 95% CIs; outcome in correlations with log markers	Individual contribution (self-rated) aggregated to the team level

**Table 2:** Process-logging schema and behavioral readouts of collaboration quality.

Field ^1^	Type	Description	Behavioral Readout (Collaboration Quality)
timestamp	ISO-8601 ^2^	Event time	pacing, latency
actor_role	Categorical	Role at event time	participation balance, role complementarity
phase_id	Categorical	Phase/stage identifier	pacing; convergence trajectory
action_type	Categorical	Create/edit/comment/vote/link	information sharing; convergence
artifact_id	String	Referenced artifact	information reuse density
dependency	String	Satisfied/blocked dependency tag	coordination latency

^1^ actor_role is coarse-grained (e.g., clinician, designer, guidance agent); artifact_id is pseudonymous and contains no content; dependency flags satisfied/blocked states; additional raw fields may exist internally but are not required for the analyses reported. ^2^ timestamps follow ISO-8601; UTC preferred.

**Table 3:** Prototype modules and responsibilities.

Module	Responsibility	I/O (Core Fields)
Session Layer	Identity, role, team, phase lifecycle	user_id, actor_role, team_id, phase_id
Guidance Prompter	Time-boxing, participation nudges, decision checkpoints	in: status; out: prompt_type, target_role, content
Workspace and Artifacts	Boards/forms/files; cross-referencing; versioning	action_type, artifact_id, link_to
Log Collector	Event capture; export to CSV/JSON; privacy filters	timestamp, actor_role, phase_id, action_type, artifact_id, dependency

**Table 4:** Operationalization and outputs (all rescaling via Equation (2); SUS via Equation (1)).

Construct	Instrument/Source	Raw Scale	Scoring/Aggregation	Output
Usability	SUS	5-point	Equation (1) → 0–100; report mean ± SD, median, IQR	S (0–100)
Usefulness	PU (TAM)	7-point	mean of rescaled items via Equation (2)	$\bar{\mathrm{PU}}\in \left[1,7\right]$
Ease of use	PEOU (TAM)	7-point	mean of rescaled items via Equation (2)	$\bar{\mathrm{PEOU}}\in \left[1,7\right]$
Team outcome	TP (experts)	rubric scores	rater-wise z, then mean, then back-scale	TP (unit-scaled)
	PP (participants)	Likert items	rescale via Equation (2), then mean	$\bar{\mathrm{PP}}\in \left[1,7\right]$
	S-TP (members)	Likert items	rescale via Equation (2), then team mean	$\bar{S-\mathrm{TP}}\in \left[1,5\right]$
Process	Logs (events)	categorical + time	counts, dispersions, latencies, reuse links	timelines, graphs

**Table 5:** Orchestrated procedure, team agreements, instruments, and data flows.

Index	Phase	Key Activities	Orchestrator Controls ^1^	Team Agreements	Instruments	Log Highlights (Examples)	Outputs
I	Orientation and Briefing	All-hands intro; goals; rules	Start gate; visible time-box; initial role assignment	Acknowledge rules; single-threaded conversation	—	Session start timestamp; phase enter; role assignment	Timeline anchor; session roster
II	Access and Onboarding	Role-based login; task materials; role-rotation checkpoint	Turn-taking prompts; role-rotation checkpoint	Follow speaking order; document ideas in DIMS	—	Login events; task-package access; initial artifact references	Access confirmation; initial links
III	Co-Creation	Guided cycles; prompts; turn-taking; artifact hand-offs	Artifact hand-off prompts; reuse highlight; within-phase timers	Handover artifacts via DIMS; reference prior notes	— (logs only)	Per-event: timestamp, role, phase, action type, artifact ID, dependency flags; sub-phase transitions	Process traces for participation balance, pacing/latency, and information reuse
IV	Sharing and Inter-Team Exchange	Summaries; brief presentation; decision	Convergence timer; decision checkpoint	Summarize options; converge within time-box	TP (experts), administered post-presentation/offline	Decision artifacts finalized; votes/decisions; dependency clearances	Decision-latency markers; artifact set for expert rating; TP entries
V	Post-Session Survey	Usability/acceptance and team-outcome scales; export	Review prompt; export artifacts	Final check; confirm completeness	SUS, PU, PEOU; PP; S-TP (participants)	Final exports; session-close timestamp	Instrument datasets; final logs

^1^ A human proctor monitored compliance only and provided no domain input.

**Table 6:** Usability and acceptance (cleaned sample, n=5).

Measure	Mean	SD	Median ^3^	IQR ^3^	95% CI ^2^	Scale Interpretation
SUS (0–100)	69.20	12.93	66.00	16.00	60.00–80.00	above common ‘good’ thresholds
PU (1–7) ^1^	4.89	1.48	4.14	2.00	3.86–6.20	moderate–high
PEOU (1–7) ^1^	5.63	1.09	5.57	1.14	4.71–6.43	high

^1^ PU/PEOU were rescaled to 1–7 per Equation (2). ^2^ 95% CIs are bias-corrected and accelerated bootstrap (BCa; B = 10,000). ^3^ Medians and IQRs are reported alongside mean ± SD due to small-N and potential non-normality; results are interpreted descriptively.

**Table 7:** Team outcomes (cleaned samples; TP n=12, PP/S-TP n=12).

Measure	Mean	Normalized Means (0–1) ^1^	SD	Median	IQR	95% CI
TP (1–5)	4.18	0.836	0.06	4.17	0.05	4.14–4.22
PP (1–7)	6.14	0.876	0.75	6.00	1.25	5.60–6.67
S-TP (1–5)	4.08	0.816	0.70	4.04	0.96	3.57–4.57

^1^ Means, SDs, medians, IQRs, and 95% CIs are computed on the raw scales (TP 1–5, PP 1–7, S-TP 1–5). The 0–1 normalized column is for visual comparison only and is not used for inference. Confidence intervals are based on team-level BCa bootstrap (B = 10,000), consistent with Section 3.2.4. Inter-rater reliability for TP (ICC(2,k) with BCa 95% CIs) is provided in Supplement Table S1.

**Table 8:** Correlation matrix (Pearson r) for conversational features and outcomes (cleaned teams) ^1^. Values are exploratory and not used for significance testing due to n=6.

	NDT	TDD	ATD	TTB	TP	PP	S-TP
NDT	1.000	0.128	−0.617	−0.437	−0.014	−0.922	−0.223
TDD		1.000	0.661	0.696	−0.278	−0.221	−0.750
ATD			1.000	0.846	−0.186	0.570	−0.264
TTB				1.000	−0.441	0.377	−0.286
TP					1.000	0.166	−0.023
PP						1.000	0.458
S-TP							1.000

^1^ Pearson correlations (r) at the team level are shown. Values are exploratory and not used for significance testing due to the small sample size (*n* = 6).

**Table 9:** Pearson correlations between conversational markers and outcomes with 95% confidence intervals (team level). Values are descriptive and not used for significance testing (*n* = 6 teams). Confidence intervals are calculated using a percentile bootstrap with a Fisher-z transformation (B = 10,000).

Marker	Outcome	Pearson r	95% CI
NDT	PP	−0.922	−1.000–−0.729
TDD	S-TP	−0.750	−0.998–−0.168
ATD	PP	0.570	−0.438–1.000
TTB	PP	0.377	−0.526–0.907

## Data Availability

The original contributions presented in this study are included in the article and Supplementary Material. Further inquiries can be directed to the corresponding author.

## Note

1 Counts are at the team level; correlations in Section 5.3 use individual-level aggregates (*n* = 12 teams).

## Supplementary Materials

The following supporting information can be downloaded at: https://www.mdpi.com/article/10.3390/bs15121704/s1, Table S1: Inter-rater reliability for Technical Performance (TP); Table S2: TP team-level mean and dispersion with BCa 95% CI (B = 10,000); Table S3: Spearman’s ρ between conversational markers (rows) and team outcomes (columns “Outcome”); Table S4: Pearson’s *r* between conversational markers and team outcomes. References (Bartko, 1966; de Winter et al., 2016; Gentle, 2010; Puth et al., 2015; C. F. J. Wu, 1986) are cited in the supplementary materials.

## Abbreviations

The following abbreviations are used in this manuscript:

PP	Perceived Performance
TP	Technical Performance
S-TP	Self-rated Technical Performance
SUS	System Usability Scale
PU	Perceived Usefulness
PEOU	Perceived Ease of Use
NDT	Number of Dialogue Turns
TDD	Total Dialogue Duration
ATD	Average Turn Duration
TTB	Turn-Taking Balance
RTC	Role-Specific Turn Counts
DSA	process-guidance controller
DIMS	shared workspace layer

## Appendix A

This appendix reproduces the item content of the three widely used instruments used to assess usability and acceptance in our field study: the System Usability Scale (SUS), Perceived Usefulness (PU), and Perceived Ease of Use (PEOU). We administer each immediately after the session and score them following established conventions. SUS is computed on a 0–100 scale using the standard odd/even transformation; PU and PEOU are aggregated as item means on a 1–7 scale after linear rescaling when required (see Equation (2) in the Section 3.

We include full item texts here to support transparency, replication, and secondary analyses. The instruments used in this article are outcome summaries of usability (SUS) and technology acceptance (PU, PEOU); detailed psychometric analyses are outside the scope of the present study.

**Table A1:** Items of System Usability Scale (SUS) (Bangor et al., 2008; Brooke, 1996).

No.	Items
SUS1	I think that I would like to use this system frequently.
SUS2	I found the system unnecessarily complex.
SUS3	I thought the system was easy to use.
SUS4	I think that I would need the support of a technical person to use it.
SUS5	I found the various functions in this system were well integrated.
SUS6	I thought there was too much inconsistency in this system.
SUS7	I would imagine that most people would learn to use this system quickly.
SUS8	I found the system very cumbersome to use.
SUS9	I felt very confident using the system.
SUS10	I needed to learn a lot of things before I could get going with this system.

**Table A2:** Items of Perceived Usefulness Scale (PU) (Davis, 1989).

No.	Items
PU1	Using this system in my job would enable me to accomplish tasks more quickly.
PU2	Using this system would improve my job performance.
PU3	Using this system in my job would increase my productivity.
PU4	Using this system would enhance my effectiveness on the job.
PU5	Using this system would make it easier to do my job.
PU6	I would find this system useful in my job.

**Table A3:** Items of Perceived Ease of Use Scale (PEOU) (Davis, 1989).

No.	Items
PEOU1	Learning to operate this system would be easy for me.
PEOU2	I would find it easy to get this system to do what I want it to do.
PEOU3	My interaction with this system would be clear and understandable.
PEOU4	I would find this system to be flexible to interact with.
PEOU5	It would be easy for me to become skillful at using this system.
PEOU6	I would find this system easy to use.

## Appendix B

Immediately after the session, we used three instruments developed in our prior work for this program: TP (expert-scored technical quality, 1–5 Likert), PP (team-level perceived performance, 1–7 Likert), and S-TP (individual self-rated technical contribution, 1–5 Likert; items parallel TP). In earlier studies, internal consistency was above a common “good” threshold (TP α = 0.953; PP α = 0.936; S-TP shares TP items and was not separately tested). Due to space limits, construction details are reported elsewhere; here, we focus on their use as outcome summaries in the pilot.

**Table A4:** Items of Technical Performance Scale (TP) (Buchanan, 1992; Clarkson et al., 2013; Cross, 1990; Norman, 2013; Pick et al., 2024; Preston & Colman, 2000; Xie et al., 2020).

No.	Items
TP1	Task Responsiveness: Does the proposal respond to the task context and functional requirements, and is the design logic coherent?
TP2	Clarity of Expression: Are the drawings clear, and are the symbols and structures easy to understand?
TP3	Originality: Does the work demonstrate novel ideas, divergent thinking, or breakthrough concepts?
TP4	Functional Feasibility: Does the solution exhibit basic medical and engineering feasibility?
TP5	Collaboration Imprint: Is there visible evidence of two-person collaboration (e.g., alternating pen traces, complementary lines of thought)?

**Table A5:** Items of Perceived Performance Scale (PP) (Mathieu et al., 2000; Preston & Colman, 2000; Woolley et al., 2010).

No.	Items
PP1	I remained actively engaged throughout the task.
PP2	Working with my partner was enjoyable.
PP3	Our communication was smooth.
PP4	I sensed mutual attunement and understanding between us.
PP5	During the collaboration, I clearly felt we were accomplishing the task together.
PP6	I am satisfied with the overall outcome of the task.
PP7	I am satisfied with my own performance.
PP8	My partner and I were broadly aligned in our design thinking.
PP9	We developed a shared path of understanding of the problem.

## Appendix C

The platform comprises a shared workspace (DIMS) and a process-guidance layer (DSA). Within the DSA, a rule-based controller evaluates “guard → action” rules and prompts the renderer to issue brief natural-language cues; connectors synchronize state and logs with DIMS. Optional creative assistants are separate and controller-agnostic.

The controller implements lightweight, rule-based orchestration that translates facilitator intent into prompts and checks aligned with conditions relevant to collective intelligence—participation balance, dependency-aware pacing, and convergence. Rules are specified as “guard → action” pairs with an optional dwell window Δ (the minimum time a condition must persist before a rule can fire), a cooldown τ (the minimum time before the same rule may fire again), and an optional priority π for conflict resolution (Kephart & Chess, 2003; Widom & Ceri, 1995), e.g., “if participation dispersion exceeds a threshold for Δ, prompt a quieter role; if a dependency is cleared but no action starts within Δ, nudge the next step.”

The workspace layer (DIMS) aggregates shared artifacts (boards, forms, files) and records cross-references, allowing for reuse and convergence to be observed without exposing content. The session layer manages identities, roles, teams, and the phase lifecycle. A log collector exports de-identified events (timestamp, role, phase, action type, artifact reference, dependency flag) to CSV/JSON with privacy filters.

The data flow is as follows: participants act in the workspace; the session layer emits states; the controller renders prompts and transitions; and events are logged for post hoc visualization (timelines, role heatmaps, reuse graphs). This appendix corresponds with Figure 2 (Section 3) and is intentionally lightweight to remain backend-agnostic and low-burden in health-system settings.

## Appendix D

The research team authored the prompts and had been using them across our program. The English versions below are author-translated and slightly refined for clarity; minor wording differences do not alter task intent.

D1. Child-Friendly Nebulizer for Home and School Use: Context. Nebulized therapy delivers atomized medication to the airways. Scenario. A five-year-old child with bronchitis struggles to use a home nebulizer independently while at school. Design brief. Design a nebulizer that is easy to operate, portable, and safe for unsupervised use by a child.

D2. Portable Digital Radiography (DR) in Crowded Wards: Context. DR systems capture X-ray images with digital detectors, but typical units are bulky and hard to maneuver. Scenario. A radiology doctor must image patients in crowded hospital spaces where positioning and device movement are difficult. Design brief. Design a portable/mobile DR unit that is compact and lightweight, enables fast imaging, and meets safety requirements.

D3. Transport-Ready Ventricular Assist System: Context. Ventricular assist devices support cardiac output and must run continuously during intra-hospital transport. Scenario. During transfers between the operating theater and ICU, complex environments cause frequent disruptions. Design brief. Design a system suitable for transport: secure fixation, orderly cable/line management, and a stable power solution.

D4. Ergonomic Blood-Flow Pump Controller: Context. Blood-flow pumps are adjusted frequently to maintain hemodynamic stability. Scenario. A clinician must rapidly change settings, but current controls have small buttons and ambiguous labels. Design brief. Redesign the pump control interface with larger buttons, clear labeling, and tactile feedback to reduce operation time and errors.

D5. Intelligent Universal Injection Tool: Context. Injection therapy (intramuscular, subcutaneous, intradermal) is routine but mode-specific. Scenario. A nurse faces decision frictions when switching between injection modes at the point of care. Design brief. Design a tool that provides mode prompts, indicates whether air expulsion is required, and automatically calibrates the dosage.

D6. Clinician Ergonomics in the Operating Theater: Context. Surgical work is prolonged and typically performed while standing; existing furniture is designed with the patient in mind. Scenario. A surgeon must stand for complex procedures with limited ergonomic support. Design brief. Design an OT ergonomic solution that allows for rapid height/angle adjustment, an optimized layout, and compliance with sterile practice.

D7. Intelligent Hand-Hygiene Management: Context. Hand hygiene is central to infection control; busy rounds reduce compliance. Scenario. A junior doctor moves between patients under time pressure, sometimes cleansing hands sub-optimally. Design brief. Design a system for real-time monitoring, rapid hand cleansing, and recording/analytics of hand-hygiene events.

D8. Inpatient Falls-Risk Management: Context. Older inpatients face an elevated falls risk, especially under certain medications. Scenario. A high-risk patient lacks consistent family supervision due to a lack of risk awareness. Design brief. Design a comprehensive falls-risk system with real-time alerts, family/caregiver notification, and education and training modules.

D9. Ostomy Bag Care and Support: Context. Post-operative ostomy care requires correct bag replacement to prevent skin injury and leakage. Scenario. A patient and family have limited time for training during hospitalization and feel uncertain about home care. Design brief. Design an easier-to-use ostomy bag together with smart guidance, a digital training platform, and remote nursing support.
