# Supplementary material for: AI-Augmented Co-Design in Healthcare: Log-Based Markers of Teamwork Behaviors and Collective Intelligence Outcomes
Source: Behav Sci (Basel). 2025 Dec 9;15(12):1704. doi: 10.3390/bs15121704 (PMC12729299; doi:10.3390/bs15121704)
Supplement: Supplementary file 1 [file behavsci-15-01704-s001.zip › behavsci-3921738-supplementary.pdf]

behavioral sciences

MDPI

Supplementary File, Behavioral Sciences

AI-Augmented Co-Design in Healthcare: Log-Based Markers of Teamwork Behaviors and Collective Intelligence Outcomes

1. Details of Uncertainty and Reliability

1.1. Collection of Computational Methods

Eq. S1. BCa confidence intervals for means (used for SUS, PU, PEOU at the individual level; PP, S-TP, TP at the team level). Let $\hat{\theta}$ be the sample mean and $\{\hat{\theta}^{*(b)}\}_{b=1}^{B}$ be the bootstrap means from resamples drawn at the corresponding level (individuals for SUS/PU/PEOU; teams for PP/S-TP/TP). Define $z_0 = \Phi^{-1}(Pr\ (\hat{\theta}^{*} < \hat{\theta}))$and the acceleration $a$from the jackknife over units (Gentle, 2010; Puth et al., 2015; Wu, 1986). The BCa limits for a two-sided $100(1-\alpha)\%$ CI are as follows:

$$\mathrm{CI}^{\mathrm{BCa}} = [\ \hat{\theta}^{*}_{(\alpha_1)}, \hat{\theta}^{*}_{(\alpha_2)}\ ], \alpha_{1,2} = \Phi\left(z_0 + \frac{z_{\alpha/2,\,1-\alpha/2}}{1 - a\, z_{\alpha/2,\,1-\alpha/2}}\right), \quad \text{(S1)}$$

Where $\Phi(\cdot)$ is the standard normal cdf (Gentle, 2010) and $z_{\alpha/2,\,1-\alpha/2}$ denotes the corresponding normal quantiles. Quantiles are taken from the bootstrap distribution and truncated to the legal scale.

Eq. S2. Inter-rater reliability for TP (ICC(2,k)). Using the two-way random-effects, absolute-agreement model with $n$teams (rows), $k$raters (columns), and ANOVA mean squares MS_R (rows), MS_C (columns), and MS_E (residual), the average-measures ICC (Bartko, 1966) is as follows:

$$ICC(2,k) = \frac{MS_R - MS_E}{MS_R + \dfrac{MS_C - MS_E}{n}}. \quad \text{(S2)}$$

Uncertainty is summarized with **BCa 95% CIs** based on team-level resampling (B = 10,000) (Puth et al., 2015). Estimates and CIs are reported in Table S1.

Eq. S3. Bootstrap CIs for correlations. For each pair (marker, outcome), compute Pearson's $r$on teams and transform via **Fisher's z**: $z = atanh\ (r)$ [61]. Draw B bootstrap resamples of teams, recompute $z^{*(b)}$, take the percentile CI $[z_{(2.5\%)}, z_{(97.5\%)}]$, and back-transform with $tanh\ (\cdot)$ to obtain the CI in r. **Spearman's ϱ** follows the same steps (with ranks) and is reported as a robustness check in Table S2 (de Winter et al., 2016).

1.2 ICC (2,k) of Technical Performance (TP)

Table S1 reports inter-rater reliability for expert ratings of Technical Performance (TP) using a two-way random-effects, absolute-agreement ICC(2,k) based on the standard ANOVA mean squares. The analysis uses the cleaned set of **n = 6 teams** and **k = 11 unique raters** (duplicate names removed). The point estimate is **negative (−0.181)** with a **BCa 95% CI of −0.408–0.013** (team-level bootstrap, B = 10,000), indicating that residual and rater-specific variation exceeded between-team variance under this small-n design. Following recommended practice, we **do not truncate** the estimate at zero; rather, we treat ICC as a diagnostic and emphasize team-level means with uncertainty intervals in the main text (Table 7). For convenience, Table S2 collates the TP mean, dispersion, and its **BCa 95% CI (4.14–4.22)**.

Table S1. Inter-rater reliability for Technical Performance (TP): ICC(2,k) with BCa 95% CIs (cleaned teams n = 6; unique raters k = 11). Mean squares are from the two-way random-effects ANOVA (rows = teams, columns = raters). CIs use team-level BCa bootstrap (B = 10,000). Negative ICC reflects greater residual/rater variance than between-team variance under sparse designs.

Statistic	Estimate	95% CI (BCa)	n_teams_used	k_raters_used	MS_R	MS_C	MS_E
ICC(2,k) for TP (absolute agreement)	-0.181	-0.408–0.013	6	11	0.0664	3.0264	0.1646

Table S2. TP team-level mean and dispersion with **BCa 95% CI** (B = 10,000). Values are on the original 1–5 scale; ranges are truncated to legal scale bounds. Reported to complement Table 7 in the main text.

Measure	Mean	SD	Median	IQR	95% CI (BCa)
Technical Performance (1–5) — mean of team means	4.18	0.06	4.17	0.05	4.14–4.22

1.3. Correlations of Pearson's r, Spearman's ρ, and their 95% CI

Tables S3–S4 summarize **team-level associations** between conversational markers and outcomes. We report both **Spearman's ϱ** (rank-based, percentile bootstrap CIs) and **Pearson's r** (percentile bootstrap with Fisher's z transform and back-transformation). Markers: **NDT** (number of dialogue turns), **TDD** (total dialogue duration), **ATD** (average turn duration), and **TTB** (turn-taking balance). Outcomes are team-level means on their original scales: **TP_std_mean** (1–5), **PP_std_mean** (1–7), **S-TP_std_mean** (1–5). With **n = 6 teams**, intervals are necessarily wide; results are interpreted **descriptively** and used to triangulate the practice-level patterns discussed in the main text (e.g., negative NDT–PP; negative TDD–S-TP; positive ATD–PP), without null-hypothesis tests.

Table S3. Spearman's ϱ between conversational markers (rows) and team outcomes (columns "Outcome"). CIs are **percentile bootstrap** at the **team level** (B = 10,000), truncated to [−1, 1]. **n_teams** = 6 for all pairs. Values are descriptive; no NHST is performed.

Marker[1]	Outcome[2]	Spearman ϱ	95% CI (percentile)[3]	n_teams
NDT	TP_std_mean	-0.257	-0.997–0.723	6
NDT	PP_std_mean	-0.928	-0.998–-0.664	6
NDT	S-TP_std_mean	-0.371	-0.998–0.759	6
TDD	TP_std_mean	-0.257	-0.952–0.664	6
TDD	PP_std_mean	-0.087	-0.940–0.997	6
TDD	S-TP_std_mean	-0.771	-1.000–0.091	6
ATD	TP_std_mean	-0.029	-0.997–0.952	6
ATD	PP_std_mean	0.522	-0.584–0.997	6
ATD	S-TP_std_mean	-0.314	-0.979–0.600	6
TTB	TP_std_mean	-0.257	-0.952–0.750	6
TTB	PP_std_mean	0.435	-0.479–0.936	6
TTB	S-TP_std_mean	-0.086	-0.965–0.822	6

[1] Marker definitions: NDT = #turns, TDD = total dialogue duration, ATD = average turn duration, TTB = turn-taking balance (closer to 1 indicates more balanced participation).
[2] "std_mean" denotes the **team mean on the original item scale** (TP 1–5; PP 1–7; S-TP 1–5).
[3] Bootstrap resampling is performed over **teams**; no imputation was used.

Table S4. Pearson's r between conversational markers and team outcomes. CIs use **percentile bootstrap on Fisher's z** with back-transformation (B = 10,000) [61]; bounds truncated to [−1, 1]. **n_teams** = 6. Results complement the r-matrix in Table 8 of the main text.

**Marker[1]**	**Outcome[2]**	**Pearson r**	**95% CI (percentile, Fisher-z)[3]**	**n_teams**
NDT	TP_std_mean	-0.014	-0.984–0.875	6

NDT	PP_std_mean	-0.922	-1.000–-0.729	6
NDT	S-TP_std_mean	-0.223	-0.996–0.876	6
TDD	TP_std_mean	-0.278	-1.000–0.503	6
TDD	PP_std_mean	-0.221	-0.936–0.984	6
TDD	S-TP_std_mean	-0.75	-0.998–0.168	6
ATD	TP_std_mean	-0.186	-0.925–0.930	6
ATD	PP_std_mean	0.57	-0.438–1.000	6
ATD	S-TP_std_mean	-0.264	-0.951–0.824	6
TTB	TP_std_mean	-0.441	-0.985–0.865	6
TTB	PP_std_mean	0.377	-0.526–0.907	6
TTB	S-TP_std_mean	-0.286	-0.952–0.800	6

[1] Marker definitions: NDT = #turns, TDD = total dialogue duration, ATD = average turn duration, TTB = turn-taking balance (closer to 1 indicates more balanced participation).

[2] “std_mean” denotes the **team mean on the original item scale** (TP 1–5; PP 1–7; S-TP 1–5).

[3] Bootstrap resampling is performed over **teams**; no imputation was used.

References

Bartko, J. J. (1966). The Intraclass Correlation Coefficient as a Measure of Reliability. *Psychological Reports*, *19*(1), 3–11. https://doi.org/10.2466/pr0.1966.19.1.3

de Winter, J. C. F., Gosling, S. D., & Potter, J. (2016). Comparing the Pearson and Spearman correlation coefficients across distributions and sample sizes: A tutorial using simulations and empirical data. *Psychological Methods*, *21*(3), 273–290. https://doi.org/10.1037/met0000079

Gentle, J. E. (2010). *Computational Statistics*. Springer Science & Business Media.

Puth, M., Neuhäuser, M., & Ruxton, G. D. (2015). On the variety of methods for calculating confidence intervals by bootstrapping. *Journal of Animal Ecology*, *84*(4), 892–897. https://doi.org/10.1111/1365-2656.12382

Wu, C. F. J. (1986). Jackknife, Bootstrap and Other Resampling Methods in Regression Analysis. *The Annals of Statistics*, *14*(4), 1261–1295. https://doi.org/10.1214/aos/1176350142
